# Estimating long-run equilibrium real exchange rates: short-lived shocks with long-lived impacts on Pakistan

**DOI:** 10.1186/2193-1801-2-292

**Published:** 2013-07-01

**Authors:** Asma Zardad, Asma Mohsin, Khalid Zaman

**Affiliations:** Department of Management Sciences, COMSATS Institute of Information Technology, Abbottabad, Pakistan

**Keywords:** Real exchange rate, Volatility, Vector error correction model, ARCH, Pakistan, JEL classification, C62, O24, O53

## Abstract

The purpose of this study is to investigate the factors that affect real exchange rate volatility for Pakistan through the co-integration and error correction model over a 30-year time period, i.e. between 1980 and 2010. The study employed the autoregressive conditional heteroskedasticity (ARCH), generalized autoregressive conditional heteroskedasticity (GARCH) and Vector Error Correction model (VECM) to estimate the changes in the volatility of real exchange rate series, while an error correction model was used to determine the short-run dynamics of the system. The study is limited to a few variables i.e., productivity differential (i.e., real GDP per capita relative to main trading partner); terms of trade; trade openness and government expenditures in order to manage robust data. The result indicates that real effective exchange rate (REER) has been volatile around its equilibrium level; while, the speed of adjustment is relatively slow. VECM results confirm long run convergence of real exchange rate towards its equilibrium level. Results from ARCH and GARCH estimation shows that real shocks volatility persists, so that shocks die out rather slowly, and lasting misalignment seems to have occurred.

**Electronic supplementary material:**

The online version of this article (doi:10.1186/2193-1801-2-292) contains supplementary material, which is available to authorized users.

## Introduction

The down fall of Bretton Wood system in 1971 obligate industrial economies to change from fixed exchange rate to a floating system. This change brought greater volatility for both the nominal and the real exchange rate (Stockman 1983; Mussa 1986). The monetary authorities were held responsible for the greater volatility of real exchange rates during the 1970s. Theoretically, Dornbusch (1976) displayed that unexpected monetary policy shocks might produce unduly large fluctuations in the exchange rates overshooting effect. Nevertheless, the hypothesis that monetary constancy was the only reason of exchange rate volatility lost ground as majority of industrial economies have stabilized inflation at annual rates lower than 3 percent. The incapability of monetary models to imitate and anticipate exchange rate fluctuations which implies that monetary volatility is only one of the some factors driving exchange rate volatility (Meese and Rogoff 1983). Policymakers strives to attain economic growth and price stability, therefore, volatile real exchange rates are connected with impulsive movements in the comparative prices in the economy. Exchange rate constancy is one of the chief factors that encourage stable economic growth, price stability and total investment in the economies. A current strand of the literature, the so-called *“New Open Economy Macroeconomics”*, argues that non-monetary factors have gained importance in explaining exchange rate volatility. They explain that productivity shocks, terms of trade shocks, government spending, trade openness and net foreign assets are very important factors in explaining real exchange rate volatility (Calderón 2004;Alam and Ahmed 2010). Serven (2002) argues that real exchange rate uncertainty is costly for the private investment in low developing countries. The high real exchange rate volatility that portrays developing economies produces an uncertain environment for investment as firm’s mark up, which reflects market power of the firm, international exposure of exports and imports, perception about future demand of goods and cost of new capital goods become difficult to forecast.

In Pakistan, an extensive increase in the volatility of exchange rates has been seen in the recent years. Exchange rate devaluation is one of the reasons that have resulted in enormous capital flight from the country which in return influences various parts of the balance of payments of a country, the balance of trade, the international competitiveness of domestic products, businesses that export and/or imports as well as the investors making international investments (Mustafa and Nishat 2004). Such a troubling situation calls for an in detail investigation of the real exchange rate determination which should direct the policy-makers to regulate exchange rate towards its stability or equilibrium path.

The economy of Pakistan performed quite well until the end of 1980s despite the major shift in policy stance by the economic managers, from a private sector led economy in the sixties to a nationalized economy in the 1970s and a shift towards liberalization, deregulation and denationalization in the decade of eighties. The period of 1980s was more focused towards denationalization and the role of public sector was to be reduced. In this context two agreements were made on Extended Fund Facility (EFF) with IMF and Structural Adjustment Loan (SAL) with World Bank. These two agreements were the preconditions of USA for rescheduling Pakistan’s $160 millions debt, accumulated over the years. Exchange Rate Policy was revised in 1982 wherein Managed Float System was adopted and Pak Rupee depreciated by 20 percent. The medium term Standby Extended Fund Facility (EFF) agreements with IMF were signed in 1988. It had conditions attached to it regarding devaluation, import liberalization, tariff reduction and financial sector reforms like deregulation of interest rate structure etc. (Ali and Tahir 2000).

It was in the decade of nineties that Pakistan recorded the lowest GDP growth in South Asia and these were also the years when Pakistan experienced a series of adjustment and stabilization reforms. Towards the end of the nineties there were some signs of improvement in macroeconomic indicators. During the period of 1990s, the exchange rate misalignment rose significantly during the period of 1990s and its volatility was very high. Besides that the current account remained high throughout the1990s, i.e. 5.9 percent of GDP compared to 2.7 percent of GDP in 1980s. The current account was largely negative due to persistent trade deficit caused by economic sanctions and substantial decline in exports. Foreign exchange reserves have never remained sufficient and hardly covered six weeks imports during 1990s (SBP 2001).

The 2000s was featured by continuation of a more liberal outward oriented economic strategy aiming at enhancing exports and to get integrated into world economy. The structural reform programs designed and initiated in 1990s, was continued and pursued during 2000s to put the economy on the path of recovery (Mahmood et al. 2008).

The current study covers detailed analysis and investigation of macroeconomic determinants of equilibrium real exchange rate and examines shocks of volatility in Pakistan. This study complements the existing evidence presented by Hyder and Mahboob (2005), where long run convergence has been found for equilibrium real exchange rate. Further, it also complements the evidence that real exchange rate volatility is highly affected by productivity differential, terms of trade, trade openness and government expenditures (see, Macdonald and Ricci 2003). The contribution of this study is to use sophisticated econometric technique i.e., ARCH, GARCH and VECM to analyze the relationship between real exchange rate volatility and its determinants over a period of 30 years i.e., 1980 to 2010 in the context of Pakistan.

The paper is organized as follows: after introduction which is presented in Section 1 above, literature review is carried out in Section 2. Data source and methodological framework is presented in Section 3. Results are discussed in Section 4 while final section concludes the study.

## Literature review

The empirical analysis of determinants of real exchange rate volatility has always been of great concern to macroeconomists. Though the area is quite unexplored in Pakistan but there exists enough international literature for determining equilibrium real exchange rate.

Macdonald and Ricci (2003) used panel data to analyze the effect of the distribution sector on the real exchange rate, while controlling the Balassa-Samuelson effect. Results show that appreciation of real exchange rate is the result of rise in productivity of distribution sector same to that of relative rise in domestic productivity of tradable does. Hau (2000) investigated the relationship between trade weighted effective exchange rate and trade openness of economy. Nonlinear inverse relationship between import share and volatility of real exchange rate is found by monetary and aggregate supply shock. Empirical analysis on a cross-section of 54 countries approves this correlation. Alterations in trade openness describe a great portion of the cross-country disparity in the instability of the effective real exchange rate. Devereux and Lane (2002) developed an empirical model of bilateral exchange rate volatility. Assumption underlying their study is that for developing countries external financial liabilities have a significant influence on preferred bilateral exchange rate volatility, while industrial countries do not face these constraints in international financial markets. Determinants of bilateral exchange rate are explored for set of countries. Results show that developing countries bilateral exchange rate volatility is negatively affected by external shocks while for industrial countries OCA variables seem additional vital and external debt is normally not substantial in clarifying bilateral exchange rate volatility.

Macdonald and Ricci (2003 () estimated long run equilibrium real exchange rate path for South-Africa. Results show that real exchange rate in early 2002 was found to be considerably more depreciated with respect to estimated equilibrium level, and the deviation of real exchange rate from equilibrium level was found to more than two years. Hviding *et al.*^2004^) employed panel data from 1986–2002 for 28 countries to studied the impact of increasing foreign exchange reserves on reducing currency volatility. Results back the suggestion that keeping sufficient reserves decreases exchange rate volatility. The effect is tough and strong; moreover, it is nonlinear and seems to work through a beckoning effect. Calderón (2004) described the real exchange rate volatility by suggesting a structural association between volatility and its determinants. Panel data for industrial and developing countries is analyzed using GMM-IV method for sample size of 1974–2003. Model employed shows that real exchange rate fluctuations are less volatile in more open countries and trade openness helps weaken the impact of volatility shocks.

Fidan (2006) employed autoregressive vectors to investigate the relationship between agricultural export, import, and the real effective exchange rate (REER) on Turkey agriculture sector. Results show that REER has significantly small impact on agricultural import and export with short duration as compared to long run. Tenreyro (2006) proposed novel approach to analyze the relationship between nominal exchange rate variability and trade flows from time period 1970–1997. Results indicate that there exists no significant relation between nominal exchange rate variability and trade flows. Benita and Lauterbach (2007) analyzed the daily volatility of the exchange rate between the U.S. Dollar and 43 other currencies from 1990–2001. Results show positive relationship among exchange rate volatility, real interest rates and intensity of central bank intervention, except for Israel which shows negative correlation. Cross country difference is reflected by positive correlations, with countries of high volatility maintain high real interest rates and employ more central bank intervention.

MacDonald and Dias (2007 () used sample size of 1988 to 2006 to evaluate effective exchange rates of 10 industrialized and market economies ranking among the top 15 contributing economies to global imbalances. Both single country and panel econometric are engaged to estimate BEER. The alterations compulsory are in the array of 27.3 to 46.6% depreciations for the Chinese Renminbi, for US dollar 5-11%, Japanese yen need 6% adjustment while no adjustment is required for Euro. Ricci *et al.*^2008^) used panel data for 48 industrial countries to estimate cointegrating link between real exchange rate and set of variables i.e., commodity terms of trade, productivity differential and external imbalances. Result shows significant positive relationship between the CPI-based real exchange rate and terms of trade, while significantly small impact of productivity growth differential between tradable and no tradable goods is found. Rise in net foreign assets and in government consumption tend to be related with rising real exchange rates.

Samara (2009) inspected the factors which determine equilibrium real exchange rate and its volatility in Syrian economy using sample from 1980–2008. Vector Error Correction Mode (VECM) and ARCH Model is used to analyze the relationship. Result shows that Syrian (RER) has been volatile around its equilibrium level in contrast, the speed of adjustment is rather slow. ARCH results displays that the real shocks volatility would continue, and the expected drop in Syrian oil production would involve a substantial decline in (RER), and in order to address the challenges of the Syrian economy a more flexible exchange rate system would be required. Kamenik and Kumhof (2009) investigated the relationship between net welfare gain of domestic price inflation over fixed exchange rate as function of trade openness and apply structural model adjusted to Chile. Net welfare gains are positive and negative for terms of trade and price rise shocks. Results show that there is negative relationship between net gain and price shocks volatility and net gains are rising in trade openness. The most significant exclusion is deeply obligated countries, where welfare gains are great for closed economies, and declining in trade openness.

Kama et al. (2012) examine the performance of GARCH family models in forecasting the volatility behavior of Pakistani FOREX market ranging from January, 2001 to December, 2009. The results f EGARCH-based evaluation of FOREX rates showed asymmetric behavior of volatility, where TARCH model showed insignificance in case of Pakistani FOREX market. Parveen et al. (2012) examines the factors contributing exchange rate vitality in Pakistan over a period 1975–2010. The result revealed that inflation is the main factor affecting exchange rate in Pakistan. The result concludes that to harmonize fiscal policies with monetary policy first and then make effective link of both these policies with trade policy. Naz et al. (2012) assesses the extent to which the movements in exchange rate affect domestic consumer prices in Pakistan by analyzing quarterly data from 1982:Q1 to 2010:Q4. The study concludes that the effect of an exchange rate shock on domestic prices is quite gradual, taking about 14 quarters to arrive at the full impact. The immediate effect of a structural one standard deviation shock to the exchange rate (which is 0.045 increase, or 4.5% appreciation) is about 0.001 (or 0.1%) decrease in the price level. This entails an impact elasticity of 0.042. The full effect of this shock, realized after about 14 quarters, is about 0.0062 (or 0.62%) decrease in the price level. This implies a dynamic pass-through elasticity of 0.137. Aftab et al. (2012) explore the impact of exchange rate volatility at sectoral level on the exports trade of Pakistan over a period of Q1 2003 to Q4 2010. The results show that exports are negatively influenced by exchange rate volatility and relative prices while positively affected by foreign income. This relationship holds for all sectors where bound testing revealed the existence of long- run relationship, although some equations results were not statistically significant. Fida et al. (2012) examine the relationship between exchange rates and external debt by utilizing quarterly data from the 1983:Q1 to 2008:Q4 period. The results suggest that there is a long-run cointegration relationship among the relevant variables in the Natrex model and there is a long run cointegration relationship between the exchange rate and external debt variables.

In appraising the above cited literature, there is a pressing need to evaluate and analyze the exchange rate volatility in the context of Pakistan. In the subsequent sections an effort has been made to empirically find out the relationship between exchange rate volatility and their key determinants in Pakistan.

## Data source and methodological framework

This study investigates the determinants of real exchange rate volatility based on determinants developed by Macdonald and Ricci (2003). The reduced form RER equation utilized in this study is presented as follows:

1 $$\mathrm{RER}*=f\;\left(\mathrm{PRO}D^{+},\mathrm{TO}T^{+},TO^{‒},\mathrm{GE}X^{‒}\right)$$

Where PROD is productivity differential (real GDP per capita relative to main trading partner), TOT is terms of trade (price of exports relative to price of imports), TO is trade openness (sum of value of exports and imports divided by nominal GDP), GEX is government expenditures (as % age of GDP).

The predictable signs of above equation are according to Elbadawi (1994); Edwards (1989); Montiel (1997) and MacDonald and Ricci (2003) i.e.,

- A rise in trade openness depreciates the RER because trade openness creates future consumption of imports which may relatively cheap; this in turn makes consumers to substitute from non-tradable to tradable goods.
- A rise in government expenditure worsen current account and depreciate real exchange rate.
- Increase in terms of trade appreciate real exchange rate, if income effect is stronger.
- Productivity differential which is explained by Balassa-Samuelson effect, describe that productivity improvements would, generally, concerted in the tradable sector and thus lead to an appreciation of real exchange rate.

ARCH and GARCH model is used to model the conditional variance, or volatility of a variable. Then residual analysis of cointegration equation is carried out to test the order of integration of the residual of Equation 1. Simple error correction model is used to estimate both short run and long run effects of explanatory time series variables. Finally Johnson cointegration test and Vector error correction is applied to determine the long-run convergence of real exchange rate towards its equilibrium level.

### Data source

The above mentioned key variables have greater theoretical importance in explaining the volatility of real exchange rate. The sample under consideration ranges from year 1980 to 2010 with 31 yearly observations. The variables used in study are described in Table 1.

**Table 1:** **Variables identification**

Variables	Definition and construction	Source
**Exchange rate measures**		
Nominal exchange rate	Bilateral nominal rate with the $US (average period normal)	WDI (2011)
Real exchange rate (RER)	Bilateral exchange rate against US$ (end period normal)	Author’s calculation
**Productivity measures**		
Real GDP per capita (PROD)	Productivity differential relative to main trading partner	WDI (2011)
**Terms of trade measure**		
Terms of trade (TOT)	Ratio of export price to import price	WDI (2011)
**Trade openness measure**		
Trade openness (TO)	Exp + IMP/GDP	WDI (2011)
**Fiscal measure**		
Government expenditures (GE)	Government expenditures (as % of GDP)	WDI (2011)

Real exchange rate is calculated on the basis of nominal exchange rate i.e., Pakistani rupee against US dollar, the only reason is that US is main trading partner of Pakistan. Among all determinants of exchange rate fluctuations, productivity differential is well known factor that explains long-run behavior of exchange rates. This concept supports Balassa-Samuelson effect, which explains that rapid economic growth is accompanied by real exchange rate appreciation because of differential productivity growth between tradable and non-tradable sectors (Drine and Rault 2001). An escalation in government spending clues to an extension in private consumption, which increase relative prices and depreciate the real exchange rate. Trade openness reflect the theoretical negative correlation with real exchange rate volatility, while a term of trade (TOT) shows positive co-movement, which imitates that any improvements in terms of trade should be connected with real appreciation in the exchange rate.

### Methodology

#### Econometric model

Comparable to all other techniques, that utilize time series data, it is essential to distinguish that unless the diagnostic tools used account for the dynamics of the link within a sequential 'causal' framework, the intricacy of the interrelationships involved may not be fully confined. For this rationale, there is a condition for utilizing the advances in time-series version. The following sequential procedures are adopted as part of methodology used.

#### Univariate test

In order to confirm the degree, these series split univariate integration properties; we execute unit root stationarity tests. The DF (Dickey & Fuller 1979 and Chambers 1981) type test is suitable testing procedures, both based on the null hypothesis that a unit root exists in the autoregressive representation of the time series.

#### ARCH and GARCH model

ARCH (Autoregressive Conditional Heteroskadasticity) model was first presented by Engle and Granger (1987). Most of statistical tools are considered for conditional mean of a random variable, but, ARCH model differ by modeling the conditional variance, or volatility of variable. In this respect we have to specify three distinct specifications, one for the conditional mean equation, one for the conditional variance, and one for conditional error distribution. Conditional mean equation (is written as a function of exogenous variables with an error term).

2 $$Y=\delta X_{t}+\in_{t}$$

3 $$\sigma_{t}^{2}=\theta +\alpha \in_{t-1}^{2}+\beta \sigma_{t-1}^{2}$$

Equation (2) shows mean equation while Equation (3) explains variance equation. Variance equation interns consists of three terms that are.

- θ is a constant term.
- $\epsilon_{t-1}^{2}$ is ARCH term which explains volatility from the previous period, measured as the lag of the squared residual from the mean equation:
- Last term $\sigma_{t‒1}^{2}$ is GARCH term and it shows last period’s forecast variance.

Conditional error in the squared volatility is given by following equation.

$$\mu_{t}=\in_{t}^{2}-\sigma_{t}^{2}$$

Substituting for the variance in the variance equation and rearranging, we can write the model in terms of the errors as:

$$\in_{t}^{2}=\theta +\left(\alpha +\beta \right)\in_{t-1}^{2}+\mu_{t}-\beta \mu_{t-1}$$

Squared error follows heteroskedastic ARMA (1,1) process. The autoregressive root which governs the persistence of volatility shocks is the sum of (α + β).

#### Error correction model

Conferring to Granger theory, binary or more integrated time series that are cointegrated have an error correction representation (Engle and Granger 1987). In this scenario the purpose of cointegration analysis is to check whether a linear arrangement of variables having unit roots is in fact stationary (Narsid Golic 2005). If this condition is satisfied then it can be said that an equilibrium association exist.

Error correction model help in estimating both short-term and long term effects of explanatory variables. ECM can be written as:

4 $$\Delta Y_{t}=\alpha \circ +\beta_{1}\Delta X_{t}-\pi \upsilon_{t-1}+Y_{t}$$

ECM has the advantage of using both short run and long run information. In this model *β*_**1**_ is the impact multiplier or short run effect that measures the immediate impact that a change in **X**_**t**_ will have on change in **Y**_**t**_ On the other hand *π* is the feedback effect or adjustment effect, and shows how much of the disequilibrium is being corrected i.e. the extent to which any disequilibrium in the previous period effects any adjustment.

#### Lag length criteria for co integration and unrestricted VAR

VAR estimation is characterized for selecting proper lag length for unrestricted VAR and co integration analysis. Lag length criteria calculates several criteria to select the lag order of an unrestricted VAR. In this regard, the estimated VAR is stable only if all roots have modulus less than one and lie inside the unit circle. If the VAR is not stable, certain outcomes (such as impulse response standard errors) are not usable Lutkepohl (1991).

#### Long run cointegration test (Johnson Cointegration)

For long run relationship Johnson cointegration is applied which include both maximum-eigen value and trace statistics. The essential condition for Johnson co integration is that all variables should be stationary at same level.

## Results and discussion

The standard Augmented Dickey-Fuller (ADF) unit root test was exercised to check the order of integration of these variables. The results obtained are reported in Table 2. Based on the ADF unit root test statistic, it was concluded that all variables are non-stationary at level, however, after taking first difference, these variables becomes stationary.

**Table 2:** **Results of unit root test**

Variable	Level	Prob. values	1^st^difference	Prob. values	Order of integration
	Constant		Constant		
**RER**	−1.5	0.51	−3.10	0.040	I(1)
**PROD**	−2.04	0.20	−7.11	0.000	I(1)
**TOT**	−1.9	0.30	−5.64	0.000	I(1)
**TO**	−2.42	0.14	−7.22	0.000	I(1)
**GEX**	−1.41	0.55	−5.98	0.000	I(1)

Note: The null hypothesis is that the series is non-stationary, or contains a unit root. The rejection of the null hypothesis is based on MacKinnon (1996) critical values i.e., at constant: -3.646, -2.954 and −2.615 are significant at 1%, 5% and 10% level respectively. The lag length are selected based on SIC criteria, this ranges from lag zero to lag four.

In next step, we divide the main output from ARCH estimation into two divisions i.e., superior part provides the standard output of the mean equation; while the inferior part, labeled “variance equation” contains the coefficients of variance equation which shown in Table 3.

**Table 3:** **ARCH model**

Dependent variable: log of real exchange rate (LRER)				
Method: ML - ARCH (Marquardt) - Normal distribution				
GARCH = C(6) + C(7)*RESID(−1)^2 + C(8)*GARCH(−1)				
**Mean equation**				
	**Coefficient**	**Std.Error**	**z-Statistic**	**Prob**
C	−3.329	13.792	−0.241	0.809
LPROD	0.791	0.2726	2.901	0.000
LTO	−0.056	0.092	−0.613	0.407
LTOT	0.508	0.091	5.534	0.000
LGEX	−0.107	0.141	−2.369	0.017
Variance equation				
C	0.996	0.090	11.044	0.000
RESID(−1)^2	−0.160	0.001	−103.234	0.000
GARCH(−1)	1.137	0.011	94.975	0.000
Statistical tests				
R-squared	0.753	Mean dependent var		45.818
Adjusted R squared	0.678	S.D. dependent var		14.240
S.E. of regression	8.075	Akaike info criterion		6.387
Sum squared resid	1499.796	Schwarz criterion		6.757
Log likelihood	−91.000	F-statistic		10.042
Durbin-Watson stat	1.973	Prob(F-statistic)		0.000

The result of Table 3 shows that ARCH and GARCH are significant in explaining volatility of real exchange rate and tend to explain internal shocks to real exchange rate. Sum of ARCH parameter that is α and GARCH parameter that is β shown in lower part of equation is very close to one, Indicating that volatility shocks of Pakistan’s real exchange rate are quite persistence. The long run coefficients of selected variables are significant as per expectations. Productivity differential (PROD) tends to explain real exchange rate movement in Pakistan, and shows a vital role in affecting the RER with a larger magnitude than other variables (Balassa-Samuelson effect). ‘PROD’ affects RER to 0.79% and explains that for every 1% increase in productivity differential is characterized by 0.79% appreciation of real exchange rate. Whereas a 1% rise in trade openness (TO) will depreciate exchange rate to 0.05%. Similarly, a negative sign of government expenditure shows inverse relation between RER and GEX. Negative sign of government expenditure explains the hypothesis that emphasis of GEX is on consumption spending and imported goods by financing with deficit. Therefore, a rise in government spending by 1% will depreciate the RER by 0.1%, so importance should be given to improve the public expenditure management. While terms of trade is positively linked with RER and explains that 1% elevation in Terms of Trade will appreciate RER by 0.5%.

### Residual analysis of cointegration equation

**Figure 1:**
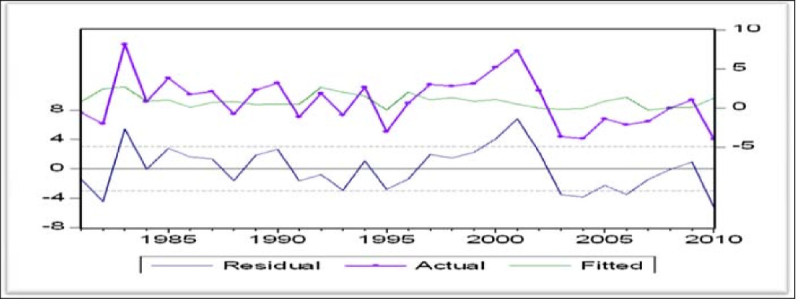
**Residual analysis.**

Cointegrated variables may float apart temporary, but must congregate systematically over time. Hence any model that levy a deterministic long-run association between a set of integrated variables, which permits those variables to diverge over the short time, would unveil cointegration relationship (Juthathip 2009). Order of integration of residual is determined by simple cointegration test. Figure 1 shows that residual is stationary which is also confirmed by unit root tests.

Figure 1 show that residual is stationary and real exchange rate for time period 1980–2010 has been volatile around its equilibrium level (fitted) in case of Pakistan. It can be seen that shocks are persistent and speed of adjustment is slow which is confirmed by ARCH results as well which explains that real shocks would correct to the equilibrium level quite slowly. From Figure 1, it can be further seen that real exchange rate is very volatile around its equilibrium level and greater misalignment has been found especially after year 2000. These fluctuations are the result of political and economic crisis occurring in Pakistan like September 9/11 incident, growing inflation, current account deficit and political crises like lawyer’s movement and militant operations in SWAT and FATA regions of Pakistan. Table 4 shows that residual are stationary at level both in ADF and Philips-Perron unit root tests.

**Table 4:** **Unit root tests for the residuals**

Estimated residual integration	Level	Mackinnon critical values for rejection of hypothesis of a unit root			Decision	Order of integration
		1%	5%	10%		
**ADF unit root test**						
Residual	−4.929	−3.670	−2.693	−2.621	Stationary at level	I (0)
**Philips-Perron (PP) unit root test**						
Residual	−4.964	−3.670	−2.693	−2.621	Stationary at level	I (0)

The residuals generated are tested for unit root to establish long-run cointegrating relationship as shown in Table 4. These residuals are stationary, which approve that above regression equation shows long-run cointegration relationship between RER and its determinants. Table 5 shows the estimation of error correction model, where ECM model is significantly explaining RER in terms of error from long run cointegration and second order lag of dependent variables (i.e. Δ(*LPROD*) and Δ(*LTO*)_*t*-2_ where Δ shows difference. Other lagged dependent variables i.e. government expenditure (LGEX) and terms of trade (LTOT) are found insignificant in explaining RER. These forces drive real exchange rate back to their long-run equilibrium levels, where these dynamics explains both short run and long run changes in RER and convergence or speed of adjustment of disequilibrium towards equilibrium adjustments in Y_t_.

**Table 5:** **Error correction mechanism**

Variable	Coefficient	Std. error	t-Statistic	Prob
C	1.450	0.489	2.965	0.006
*ϵ*_*t*-1_	−0.318	0.119	−2.678	0.013
Δ(LPROD)_*t*-2_	0.729	4.568	1.779	0.087
Δ(LTO)_*t*-2_	−0.511	3.942	2.041	0.052
**Statistical tests**				
R-squared	0.561	Mean dependent var		0.900
Adjusted R-squared	0.538	S.D. dependent var		8.521
S.E. of regression	2.450	Akaike info criterion		6.773
Sum squared resid	144.135	Schwarz criterion		6.867
Log likelihood	−99.606	F-statistic		15.851
Durbin-Watson stat	2.177	Prob(F-statistic)		0.000
**Residual tests:**				
Jarque-Bera test		0.903 (0.636)		
ARCH test		0.220 (0.803)		
White Heteroskedasticity		0.569 (0.188)		
Breusch-Godfrey serial correlation LM test		1.324 (0.117)		
**Stability tests:**				
Ramsey RESET test		2.027(0.165)		

Dependent variable: Δ(LRER).

Dynamic equation shows that ECM term correct the disequilibrium of system (see, Table 5). ECM term which measures the speed of adjustment towards equilibrium is negative and significant which shows convergence towards equilibrium level in long run. Loading factor (error correction term), indicates that convergence process would converge each year for 31.8%. Results show gradual convergence of real exchange rate in long run.

**Figure 2:**
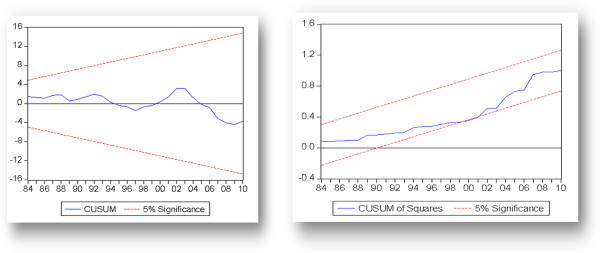
**CUSUM and CUSUM square test.**

Stability test via CUSUM and CUSUM square is shown in Figure 2. The results explains that the coefficient of Equilibrium real exchange rate are not stable over time, where the stability is a requirement for using this model for out sample forecasting. Results backend the outcome from variance equation of ARCH estimates that shock volatility is persistence in Pakistan economy.

Further, lag length criteria indicates that, the lag one is the appropriate lag order for the unrestricted stable VAR (Schwarz information criterion test) which is shown in Table 6.

**Table 6:** **Roots of characteristic polynomial**

Roots of characteristic polynomial	
Lag specification: 1 1	
Root	Modulus
0.954	0.954
0.838	0.838
0.584 – 0.268i	0.643
0.584 + 0.268i	0.643
−0.209	0.209
No root lies outside the unit circle.	
***VAR satisfies the stability condition.***	

In this study, all variables are found stationary at 1^st^ difference which provides a rationale for Johansen cointegration. As, results are significant and cointegration equation exists then it provide ladder for employing vector error correction but if there exists no relationship then we move to estimate VAR. Table 7 shows results of Johnson cointegration.

**Table 7:** **Johansen’s test for multiple cointegration vectors**

H0:	H1:	Test	0.05 Critical	Prob. **
		Statistics	Values	
λtrace		Λtrace		
*r*=0*	*r*>0	100.250	88.803	0.005
*r*≤1	*r*>1	57.500	63.876	0.153
*r*≤2	*r*>2	32.093	42.915	0.383
*r*≤3	*r*>3	13.002	25.782	0.738
*r*≤4	*r*>4	4.975	12.517	0.599

Note: Trace test indicates 1 cointegrating equations at the 0.05 level, * denotes rejection of the hypothesis at the 0.05 level.**MacKinnon-Haug-Michelis (1999) p-values.

Results from Johansen cointegration proved the existence of cointegrating equation. Trace test confirms that there exists one cointegrating equation at 5% level which shows that long run relationship exists among variables. Approximating the numerical long-run relationship among the real exchange rate, the determinants and short-run variables, this is equivalent to estimating a reduced form real exchange rate model. This is normally achieved using a VECM approach. Johnson cointegration test indicates that there exists one cointegration equation which eliminates the use of VAR model. So we move on to use VECM in order to determine long run relationship of equilibrium real exchange rate. Table 8 shows the estimates of vector error correction model.

**Table 8:** **Vector error correction model**

Cointegrating eq:	CointEq1				
LOG(RER(-1))	1.000				
LOG(PROD(-1))	-1.330				
	(0.126)				
	[-10.534]				
LOG(TOT(-1))	-0.260				
	(0.064)				
	[-4.076]				
LOG(TO(-1))	0.114				
	(0.052)				
	[2.168]				
LOG(GEX(-1))	0.457				
	(0.049)				
	[ 9.189]				
C	-4.374				
	(0.154)				
	[-28.301]				
Error correction:	Δ (LOG(RER))	Δ (LOG(PROT))	Δ (LOG(TOTE))	Δ (LOG(TOFE))	Δ (LOG(GEX))
CointEq1	-0.866	-0.019	-0.099	0.031	-0.013
	(0.022)	(0.040)	(0.063)	(0.061)	(0.075)
	[-38.913]	[-0.492]	[-1.567]	[ 0.510]	[-0.176]

Note: Standard errors in ( ) & t-statistics in [ ].

From the results of VECM, it is confirmed that long term parameters are statistically significant and consistent with the previous literatures. Relative productivity (the largest magnitude) ,terms of trade are related with more appreciation in RER; while in contrast the government expenditure and trade openness are associated with depreciation in RER. Error correction term which measures the speed of adjustments towards equilibrium should have negative sign for convergence. From results it can be seen that error correction term is significant and has right sign (negative sign). This indicates convergence towards equilibrium level. Table 9 explained short run and long run dynamics.

**Table 9:** **Short and long-run dynamics of the system**

Cointegrating eq:	CointEq1				
LOG(RER(-1))	1.000				
LOG(PROD(-1))	-1.330				
	(0.126)				
	[-10.534]				
LOG(TOT(-1))	-0.260				
	(0.064)				
	[- 4.076]				
LOG(TO(-1))	0.114				
	(0.052)				
	[2.168]				
LOG(GEX(-1))	0.457				
	(0.049)				
	[ 9.189]				
C	-4.374				
	(0.154)				
	[-28.301]				
Error correction:	D(LOG(RER))	D(LOG(PROD))	D(LOG(TOT))	D(LOG(TO))	D(LOG(GEX))
CointEq1	-0.866	-0.019	-0.099	0.031	-0.013
	(0.022)	(0.040)	(0.063)	(0.061)	(0.075)
	[-38.913]	[-0.492]	[-1.567]	[ 0.510]	[-0.176]
D(LOG(RER(-1)))	-0.067	0.009	0.134	-0.008	0.009
	(0.017)	(0.031)	(0.048)	(0.047)	(0.058)
	[-3.925]	[ 0.304]	[ 2.737]	[-0.169]	[ 0.165]
D(LOG(PROD(-1)))	-0.611	-0.288	-0.144	-0.291	-0.066
	(0.125)	(0.228)	(0.354)	(0.350)	(0.425)
	[-4.859]	[-1.261]	[-0.40286]	[-0.832]	[-0.156]
D(LOG(TOT(-1)))	0.067	0.073	0.002	-0.028	-0.066
	(0.070)	(0.127)	(0.200)	(0.195)	(0.238)
	[0.956]	[ 0.571]	[ 0.014]	[-0.143]	[-0.278]
D(LOG(TO(-1)))	0.270	0.043	0.103	0.091	0.114
	(0.088)	(0.159)	(0.251)	(0.245)	(0.298)
	[ 3.073]	[ 0.271]	[ 0.410]	[ 0.372]	[ 0.385]
D(LOG(GEX(-1)))	0.170	-0.002	-0.252	-0.351	-0.479
	(0.069)	(0.126)	(0.198)	(0.193)	(0.235)
	[ 2.454]	[-0.018]	[-1.273]	[-1.813]	[-2.036]
R-squared	0.992	0.103	0.296	0.174	0.188
Adj. R-squared	0.991	-0.120	0.121	-0.032	-0.014
F-statistic	556.314	0.463	1.688	0.842	0.927

Note: Standard errors in ( ) & t-statistics in [ ].

## Summary and conclusion

The objective of this study is to explore the factors which affect the volatility of real exchange rate in Pakistan’s economy and determine the equilibrium real exchange rate (RER). The study uses two estimation techniques i.e., Vector Error Correction Mode (VECM) and ARCH Model. We estimated the model of real equilibrium exchange rate that involved the main theoretical factors which have a real significant in the regression analysis. Based on theoretical literature there are almost four important factors causing real exchange rate volatility i.e., relative productivity, government expenditure, term of trade and trade openness. Estimation approach gives important results which are given as following:

First, there is a solid evidence that relative productivity (positive effect) (the well-known Balassa-Samuelson), terms of trade (positive effect), government expenditure (negative effect) and trade openness (negative effect) are reflected as the most driving factors of the Pakistan’s real exchange rate, which approve the hypothetical relations between the real exchange rate volatility and its determinates. Government expenditure results in depreciation of real exchange rate which indicates the inefficiency of government spending mostly in tradable sector as emphasized by literature. Real exchange rate of Pakistan has been found volatile around its equilibrium level for whole period from 1980–2010. ARCH results shows that real shocks volatility will be persistence, so that shocks die out relatively slowly, and lasting misalignment seem to have occurred.

Second, the sign of error correction term is negative as expected; it shows convergence to equilibrium level in long-run. Significant error correction term indicates speed of adjustment to equilibrium level relatively slow and it is also confirmed by error term of VECM. On the contrary, Stability tests designate that the coefficients of this dynamic real exchange rate equation are not stable over time, where the stability is essential for using this model for out sample forecasting. This outcome ratifies that the shock volatility is persistence, similarly to that in the variance equation in the ARCH equation. Policy recommendation derived from the study is that more flexible exchange rate system should be used as it would result in convergence towards equilibrium level and has fewer misalignments then fixed exchange rate. A stable exchange rate could be an effective policy instrument for promoting exports of Pakistan. However, to get further insight we need to investigate the impact of these determinants of export growth at disaggregate level. Pakistan economy needs more deep analysis to be done in this respect, and regular re-estimation of the equilibrium real exchange rate to discover potential current and future volatility.

## Authors’ original submitted files for images

Below are the links to the authors’ original submitted files for images. Authors’ original file for figure 1 Authors’ original file for figure 2
