# Supplementary figures and images for: Estimating long-run equilibrium real exchange rates: short-lived shocks with long-lived impacts on Pakistan
Source: Springerplus. 2013 Jul 1;2(1):292. doi: 10.1186/2193-1801-2-292 (PMC3706721; doi:10.1186/2193-1801-2-292)

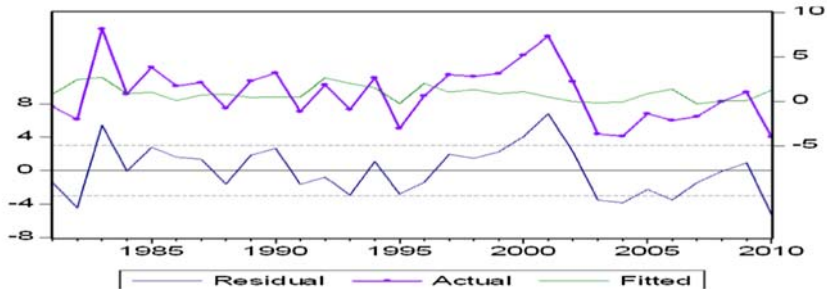

Supplement: Supplementary file 1 — Authors’ original file for figure 1 [file 40064_2013_360_MOESM1_ESM.pdf]

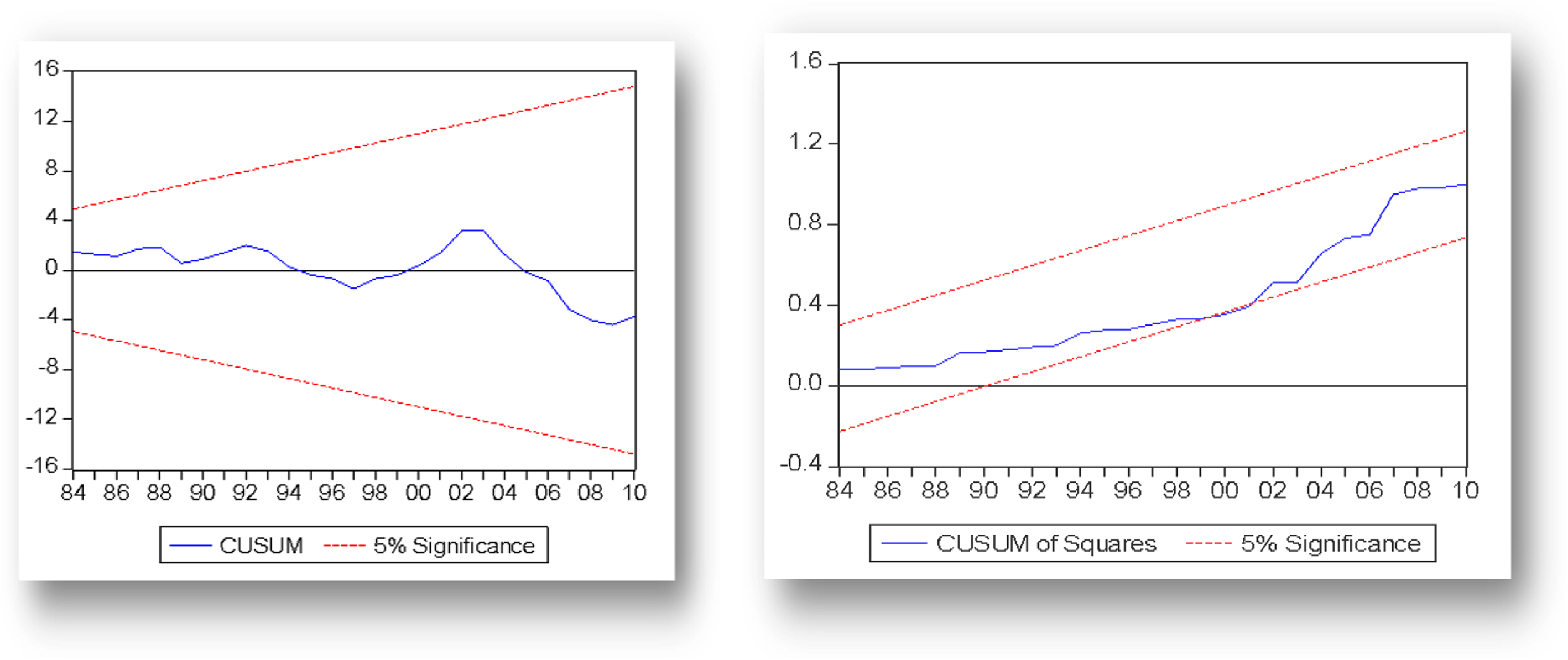

Supplement: Supplementary file 2 — Authors’ original file for figure 2 [file 40064_2013_360_MOESM2_ESM.tif]
